# Inhibition of Rat 5*α*-Reductase Activity and Testosterone-Induced Sebum Synthesis in Hamster Sebocytes by an Extract of *Quercus acutissima* Cortex

**DOI:** 10.1155/2015/853846

**Published:** 2015-01-29

**Authors:** Junichi Koseki, Takashi Matsumoto, Yosuke Matsubara, Kazuaki Tsuchiya, Yasuharu Mizuhara, Kyoji Sekiguchi, Hiroaki Nishimura, Junko Watanabe, Atsushi Kaneko, Tomohisa Hattori, Kazuya Maemura, Yoshio Kase

**Affiliations:** ^1^Tsumura Research Laboratories, Tsumura & Co., 3586 Yoshiwara, Ami-machi, Inashiki-gun, Ibaraki 300-1192, Japan; ^2^Kampo Formulations Development Center, Tsumura & Co., 3586 Yoshiwara, Ami-machi, Inashiki-gun, Ibaraki 300-1192, Japan

## Abstract

*Objective*. *Bokusoku* (BK) is an extract from the *Quercus* cortex used in folk medicine for treatment of skin disorders and convergence, and is present in *jumihaidokuto*, a traditional Japanese medicine that is prescribed for purulent skin diseases like acne vulgaris. The excess of sebum production induced by androgen is involved in the development of acne. Our aim is to examine whether BK and its constituents inhibit testosterone metabolism and testosterone-induced sebum synthesis. *Methods*. Measurements of 5*α*-reductase activity and lipogenesis were performed using rat liver microsomes and hamster sebocytes, respectively. *Results*. BK dose-dependently reduced the conversion of testosterone to a more active androgen, dihydrotestosterone in a 5*α*-reductase enzymatic reaction. Twenty polyphenols in BK categorized as gallotannin, ellagitannin, and flavonoid were identified by LC-MS/MS. Nine polyphenols with gallate group, tetragalloyl glucose, pentagalloyl glucose, eugeniin, 1-desgalloyl eugeniin, casuarinin, castalagin, stenophyllanin C, (−)-epicatechin gallate, and (−)-epigallocatechin gallate, inhibited testosterone metabolism. In particular, pentagalloyl glucose showed the strongest activity. BK and pentagalloyl glucose suppressed testosterone-induced lipogenesis, whereas they weakly inhibited the lipogenic action of insulin. *Conclusions*. BK inhibited androgen-related pathogenesis of acne, testosterone conversion, and sebum synthesis, partially through 5*α*-reductase inhibition, and has potential to be a useful agent in the therapeutic strategy of acne.

## 1. Introduction

Hormonal imbalance plays a critical role in the incidence and/or development of acne vulgaris. Androgen stimulates proliferation of sebocyte and synthesis of sebum in the sebaceous follicles. Its excess is thought to be one of causes of acne [1–3]. Testosterone is a steroid hormone from the androgen group and secreted primarily by the testicles and ovaries. The enzyme steroid 5*α*-reductase catalyzes the conversion of testosterone to a more active androgen, 5*α*-dihydrotestosterone (DHT). There are three isoenzymes of 5*α*-reductase (5*α*-R1, 5*α*-R2, and 5*α*-R3), which vary in different tissues with age. In adult skin, the 5*α*-R1 isoform is expressed dominantly, and sebocytes in the sebaceous follicles and epidermal keratinocytes are reported to express the 5*α*-R1 isoform of the enzyme [4–7].

Several studies addressed phytotherapy for acne vulgaris, and some papers implied a pharmacological mechanism depending on inhibition of 5*α*-reductase activity [8]. Gallate polyphenols, like epicatechin gallate, epigallocatechin gallate, theaflavin digallate, and pentagalloyl glucose, are reported to inhibit enzymatic reaction of the 5*α*-R1 isoform but not 5*α*-R2, while isoflavones like genistein and kaempherol preferentially inhibit the 5*α*-R2 isoform [9–12]. Moreover, it is revealed that the presence of a catechol group actuates selectivity for the 5*α*-R1 isoform [13]. In clinical studies, a green tea extract that is rich in catechins led to a reduction of skin sebum production in healthy volunteers [14], and epigallocatechin gallate significantly improved acne pathology [15]. These phytomedicines have various host-defense effects including anti-inflammatory, antioxidant, and antiapoptotic effects, thereby providing a therapeutic rationale for the use of phytomedicines in acne [8, 16].

Oak is the common name for trees in the genus* Quercus* of the beech family Fagaceae and is rich in gallate polyphenols and catechins. Oak wood is familiar in western countries and used widely for wooden materials, wine and spirit barrels, smoking chips, tanning leather, and the like. On the other hand, in eastern Asia,* Quercus acutissima*, a tree of the genus* Quercus* that lives mainly in the Asian region, has been used in folk medicine for treatment of skin disorders and convergence. In Japan, a hot water extract of the cortex of* Quercus acutissima* or its allied species is called* bokusoku* (BK) and is present in* jumihaidokuto*, a pharmaceutical-grade traditional Japanese medicine that has been widely used for the treatment of various skin disorders including acne [17, 18]. Several studies have identified constituents of BK, showing that BK contains various types of gallate polyphenols and catechins [19–23].

There is no report showing how BK improves skin disorders and what constituents contribute to BK's effect. In the present study, we addressed whether BK affects 5*α*-reductase activity and testosterone-induced sebum synthesis, which are important factors in the pathogenesis of acne vulgaris and tried to identify their active constituents by bioassay and quantitation techniques using liquid chromatography-mass spectrometry with tandem mass spectrometry (LC-MS/MS).

## 2. Materials and Methods

### 2.1. Test Samples

BK extract in the form of a dried powder (lot number 2131093010) was obtained from Tsumura & Co. (Tokyo, Japan), which manufactures it as an aqueous extract from the cortex of* Quercus acutissima*. BK was suspended at 30–100 mg/mL in dimethyl sulfoxide with sonication for 10 min and then used for assays. In cell culture assays, BK was added to the culture medium after filtering diluted BK through a 0.45 *μ*m membrane.

As candidate compounds of BK constituents, 1,2,3,6-tetra-O-galloyl glucose (tetragalloyl glucose) and 1,2,3,4,6-penta-O-galloyl glucose (pentagalloyl glucose) with purities high enough to be evaluated in biological tests were isolated from* Paeonia lactiflora* at Tsumura & Co. [24]. Similarly, eugeniin, 1-desgalloyl eugeniin, casuarinin, castalagin, and stenophyllanin C were extracted from* Quercus stenophylla* at Tsumura & Co. [25]. Gallic acid, methyl gallate, (+)-catechin, (−)-epicatechin gallate, (−)-gallocatechin, (−)-epigallocatechin gallate, quercetin, fraxin, chrysin, luteolin, and genistein were purchased from Wako Pure Chemical Industries (Osaka, Japan). Quercitrin and 2′,5-di-O-galloyl hamamelose (hamamelitannin) were purchased from Sigma-Aldrich (St. Louis, MO). (+)-Gallocatechin (Nacalai Tesque, Kyoto, Japan) and (+)-taxifolin (Extrasynthese, Lyon, France) were also purchased for this study. TMF-4AS-1, 5*α*-reductase inhibitor was purchased from Sigma-Aldrich as a positive control of an enzyme assay.

### 2.2. Preparation of Rat Liver Microsomes

Rat liver microsomes were prepared by the method of Lee et al. [12]. Male Sprague–Dawley rats (body weight, 300 to 320 g; Clea Japan, Tokyo, Japan) were fasted overnight to decrease the concentration of liver glycogen and then sacrificed. The liver was excised and washed in ice-cold homogenizing buffer containing 0.32 mol/L sucrose, 1 mmol/L dithiothreitol (Wako Pure Chemical Industries), and 20 mmol/L potassium phosphate, pH 6.5. The following procedures were all carried out at 4°C. The tissue was minced with a pair of scissors and then homogenized in homogenizing buffer using a Potter-Elvehjem grinder. The homogenate was then centrifuged at 10 000 g for 10 min. The resulting pellet was washed with two pellet volumes of homogenizing buffer and centrifuged again. The supernatants from the two centrifugations were combined and further centrifuged at 105 000 g for 1 h. The resulting pellet (microsomes) was suspended in homogenizing buffer and centrifuged again. The microsomes were resuspended in homogenizing buffer. The microsome suspension was divided into small aliquots and stored at −80°C. The microsomes were diluted with 40 mmol/L potassium phosphate, pH 6.5, immediately before use. Unlike human liver, rat liver microsomes have the 5*α*-R1 but not the 5*α*-R2 isoform.

### 2.3. 5*α*-Reductase Assay Using Rat Liver Microsomes

Briefly, the reaction solution was preincubated with or without a test sample in 1 mmol/L dithiothreitol, 40 mmol/L potassium phosphate, pH 6.5, 100 *μ*mol/L NADPH (Oriental Yeast, Tokyo, Japan), and 3.5 *μ*mol/L testosterone (Wako Pure Chemical Industries) in a final volume of 0.5 mL for 20 min at room temperature. The reaction was started with the addition of liver microsomes (20 *μ*g protein). After incubation at 37°C for 30 min, the reaction solution was extracted with 2 mL ethyl acetate. The ethyl acetate phase (upper phase) was transferred to a tube and evaporated using a centrifugal concentrator (Tomy Seiko, Tokyo, Japan). Testosterone and the metabolites were measured by two chromatography methods, HPLC (Shimadzu, Kyoto, Japan) and TLC.

The residue was dissolved in acetonitrile and analyzed using the HPLC system. The reverse-phase HPLC analysis was performed on a C18 column (3.0 × 150 mm, GL Science, Japan) at a column oven temperature of 50°C and UV detection of 240 nm. The isocratic mobile phase of acetonitrile/water (50 : 50, v/v) was flowed at 0.4 mL/min through the column.

To detect the steroids by TLC, the residue containing steroids was taken up in ethanol and chromatographed on a TLC plate (Silica gel 60 F_254_, Merck, Darmstadt, Germany), using ethyl acetate-hexane (1 : 1, v/v) as the developing solvent system at room temperature. Testosterone, (Tokyo Chemical Industry Co., Tokyo, Japan) and 5*α*-androstane-3*α*,17*β*-diol (Sigma-Aldrich) were applied to TLC as androgen standards. The plate was exposed to 10% sulfuric acid using an atomizer and then heated on a hot plate. The fluorescent signals were visualized by irradiation of UV light at 302 nm using a ChemiDoc XRS Plus (BIO-RAD, Hercules, CA).

### 2.4. Sebum Synthesis Assay

Primary hamster sebocytes (Ha-SE) were purchased from Kurabo Industries (Osaka, Japan) and maintained in a culture medium containing 10 *μ*g/mL epidermal growth factor (EGF), 2% (v/v) human serum (HS), 8% (v/v) heat-inactivated fetal bovine serum (FBS) (Kurabo Industries), and a cocktail of penicillin G and streptomycin (Sigma-Aldrich). All assays using Ha-SE cells were performed between the 5th and 6th passage. For the study dealing with sebum synthesis, a sebum detecting kit (Kurabo industries) was used. The Ha-SE cells were precultured in 24-well plates until confluence was reached and then treated every 2 d with a test sample in the presence of 10 *μ*mol/L testosterone, 10 *μ*g/mL insulin, or the vehicle in culture medium supplemented with 2% HS and 8% FBS for up to 8 days. The amount of sebum synthesized was evaluated by measuring lipid production and cell growth according to the manufacturer's instructions. That is, the lipid in sebaceous cells was stained with Oil Red O, extracted in 100% isopropanol, and quantified by a spectrometer at 520 nm. The cell growth was measured using XTT reagent (Biological Industries, Beit Haemek, Israel). The lipogenesis was calculated following the formula as relative sebum synthesis per cell number: lipogenesis = *A*/*B*, where *A* = OD 520 nm of 200 *μ*L extract solution of 300 *μ*L 100% isopropanol for extract of Oil red O dye and *B* = OD 465/630 nm of 200 *μ*L of 450 *μ*L culture fluids containing XTT reagent following incubation for 30 min. All images were taken at ×20 magnification using Biorevo BZ-9000 (KEYENCE, Osaka, Japan) immediately after staining the cells with Oil Red O.

### 2.5. Measurement of Constituents in BK by LC-MS/MS

BK powder (100 mg) was suspended in 4 mL of methanol/purified water (75 : 25, v/v). The suspension was shaken and sonicated for 5 min each. After centrifugation at 1700 g for 5 min at room temperature, the supernatant was collected as the first extract solution. Then, 4 mL of methanol/purified water (50 : 50, v/v) was added to the residue, and the second extraction solution obtained by the procedure described above was mixed with the first extract solution. The mixed extract solution undiluted or diluted 10- or 100-fold with methanol was injected into the LC-MS/MS system after pooling with a respective internal standard solution, niflumic acid (Sigma-Aldrich), or vincamine (Tokyo Chemical Industry Co.). Two systems were used for analysis of the constituents: system 1, an API4000 triple quadrupole mass spectrometer (AB SCIEX, Tokyo, Japan) equipped with an Agilent 1100 system (Agilent Technologies, Inc., Tokyo, Japan); system 2, a TripleQuad6500 (AB SCIEX) equipped with an Agilent 1290 system (Agilent Technologies, Inc.). Analytical conditions are summarized in the Supplementary Tables S1 and S2 (available online at http://dx.doi.org/10.1155/2015/853846).

### 2.6. Statistical Analysis

All values are expressed as the mean ± SEM. Statistical significance was evaluated by one- or two-way analysis of variance (ANOVA), and a probability of less than 0.05 was considered significant on the Dunnett's test (∗) or Student's *t*-test (#).

## 3. Results

### 3.1. Inhibition of Rat Liver Microsomal 5*α*-Reductase Activity by BK Extract

**Figure 1:**
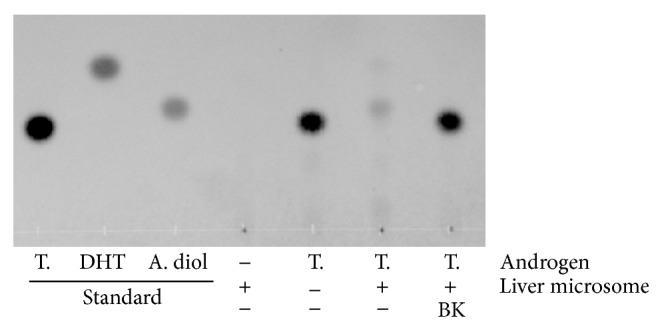
Representative TLC-image showing inhibition of rat liver microsomal 5*α*-reductase in testosterone metabolism by BK treatment. Testosterone (T.) was incubated at a final concentration of 3.5 *μ*mol/L for 30 min in the presence or absence of rat liver microsomes (40 *μ*g/mL) and the cofactors.* Bokusoku* (BK) was added at 30 *μ*g/mL before starting the reaction. The androgens were extracted with ethyl acetate after the incubation and analyzed by TLC. All androgen standards were applied at 0.1 *μ*g/*μ*L/spot. DHT: dihydrotestosterone, A. diol: 5*α*-androstane-3*α*,17*β*-diol.

We first evaluated the effects of BK in a 5*α*-reductase assay using rat liver microsomes. In this assay, testosterone added to the enzymatic reaction as a substrate was metabolized to DHT by 5*α*-reductase, followed by the subsequent conversion to 5*α*-androstane-3,17-diol by aldo-keto reductase. As shown in Figure 1, a representative TLC image, exogenous testosterone was dramatically decreased in the control. In addition, 5*α*-androstane-3,17-diol was found at a position of Rf 0.243, while the androgen standards testosterone, 5*α*-androstane-3,17-diol, and DHT appeared at Rf 0.224, 0.243, and 0.342, respectively. In contrast, the spot of testosterone treated with 30 *μ*g/mL BK was not changed compared with that of testosterone alone without microsomes, showing that BK strongly inhibited the metabolism of testosterone.

**Figure 2:**
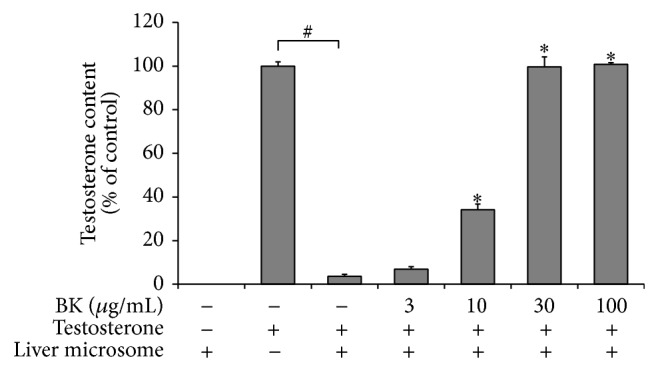
Quantification of inhibitory effect of BK on rat liver microsomal 5*α*-reductase activity. Testosterone was incubated at a final concentration of 3.5 *μ*mol/L for 30 min in the presence or absence of rat liver microsomes (40 *μ*g/mL) and the cofactors.* Bokusoku* (BK) was added to the reactions at 3, 10, 30, or 100 *μ*g/mL before starting the reaction. Testosterone remaining was extracted with ethyl acetate after the incubation and quantified by HPLC technique. Data are shown as relative amounts of testosterone, which are a percentage of testosterone alone control reaction. *N* = 3.

Next, testosterone metabolism in the same enzymatic assay was quantified by HPLC (Figure 2). BK inhibited 5*α*-reductase activity at concentration-dependent manner, and the IC_50_ was 12.2 *μ*g/mL. Moreover, 5*α*-reductase inhibitor TMF-4AS-1 also inhibited testosterone metabolism by 50% at 0.01 *μ*mol/L and higher (data not shown).

### 3.2. Suppression of Sebum Production in Hamster Primary Cells by BK Extract

**Figure 3:**
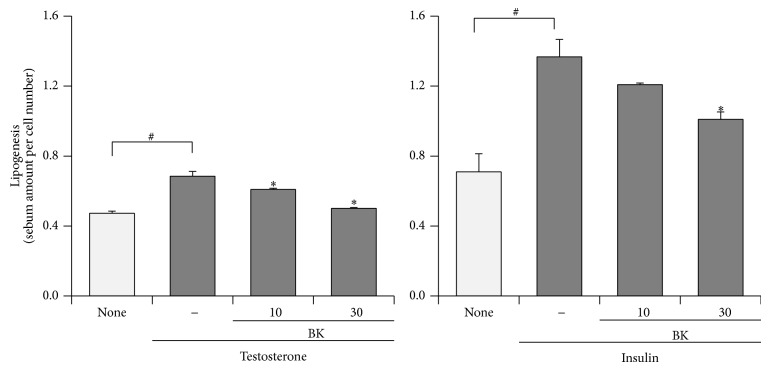
Suppression of testosterone-induced sebum synthesis of hamster primary sebocytes by BK treatment. Hamster-derived sebaceous gland cells (Ha-SE) were precultured, until confluent in the presence of epidermal growth factor, and further treated with the indicated concentrations of* bokusoku* (BK) and a differentiation factor, 10 *μ*mol/L testosterone or 10 *μ*g/mL insulin. Eight days later, the sebum synthesis was determined using a lipogenesis detecting assay kit. *N* = 3.

**Figure 4:**
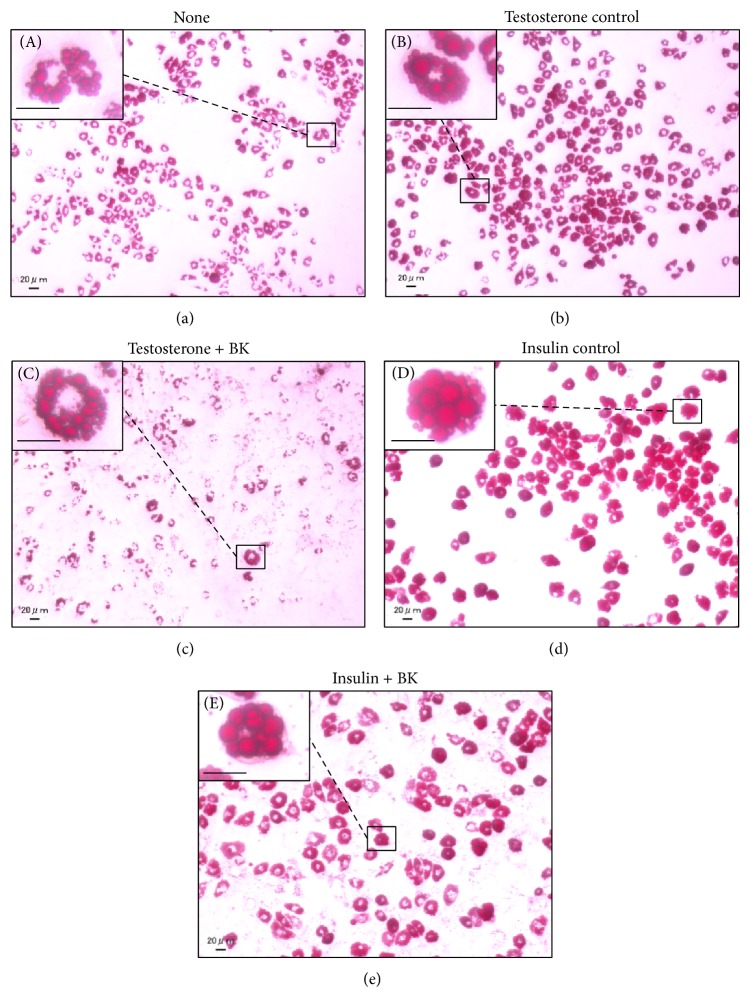
Representative images of hamster primary sebocytes treated with or without BK in the presence of a differentiation factor. Hamster-derived sebaceous gland cells (Ha-SE) were precultured until confluent in the presence of epidermal growth factor, and further treated with 10 *μ*g/mL* bokusoku* (BK) or vehicle in the presence of a differentiation factor, 10 *μ*mol/L testosterone or 10 *μ*g/mL insulin. Eight days later, cells were stained with Oil Red O. The representative images of sebum droplets were shown for ((A), (a)) none, ((B), (b)) testosterone + vehicle, ((C), (c)) testosterone + BK, ((D), (d)) insulin + vehicle, and ((E), (e)) insulin + BK. Scale bars: 20 *μ*m. The images shown in (A), (B), (C), (D), and (E) were enlargements of those shown in (a), (b), (c), (d), and (e), respectively.

To confirm that BK can regulate sebum synthesis, BK was evaluated in testosterone- or insulin-induced lipogenesis assays using Ha-SE cells. As shown in Figure 3, testosterone and insulin increased sebaceous lipogenesis. BK tested at 10 and 30 *μ*g/mL significantly suppressed testosterone-induced sebum accumulation. On the other hand, BK suppressed the insulin-induced lipogenesis at 30 *μ*g/mL with a lower inhibition compared with the action in testosterone-induced lipogenesis. Moreover, BK dramatically decreased number of sebum-producing cells 8 days after stimulating with the differentiation factor, especially testosterone (data not shown); however, BK did not exert a cytotoxic activity against undifferentiated Ha-SE cells (Supplementary Figure S1). Representative images in Figure 4 show that BK impeded the formation of sebum at 10 *μ*g/mL in sebocytes treated with testosterone.

### 3.3. Measurement of Constituents in BK by LC-MS/MS

There are several reports identifying the constituents of BK [19–23], but no report of a quantitative study. Compounds marked with an asterisk in Table 1 have been already reported to exist in BK. Considering the literature on compounds in other plants related to* Quercus acutissima* and active polyphenols to inhibit 5*α*-reductase activity, a total of 21 compounds in Table 1 were measured. As a result, most of the candidate compounds were detected. Pentagalloyl glucose, eugeniin, 1-desgalloyl eugeniin, castalagin, stenophyllanin C, (±)-gallocatechin, gallic acid, and (+)-catechin were present at more than 1 mg/gram extract and were abundant compared with the others. In addition, amounts of tetragalloyl glucose, hamamelitannin, casuarinin, methyl gallate, (−)-epicatechin gallate, (−)-epigallocatechin gallate, fraxin, (+)-taxifolin, luteolin, quercetin, quercitrin, and genistein were quantified in BK, although chrysin was below the quantification limit.

### 3.4. Identification of Active Constituents

Focusing on the 17 main constituents found in BK, a 5*α*-reductase assay using rat liver microsomes was performed to identify the active constituents of BK (Table 2). All test samples were examined at 0.1, 1, 10, and 100 *μ*mol/L. Tetragalloyl glucose, pentagalloyl glucose, eugeniin, 1-desgalloyl eugeniin, casuarinin, castalagin, stenophyllanin C, (−)-epicatechin gallate, and (−)-epigallocatechin gallate actively inhibited 5*α*-reductase activity by more than 50% at 10 and 100 *μ*mol/L. On the other hand, hamamelitannin, (+)-catechin, (+)-gallocatechin, (−)-gallocatechin, flaxin, gallic acid, (+)-taxifolin, and methyl gallate did not show any effect.

**Figure 5:**
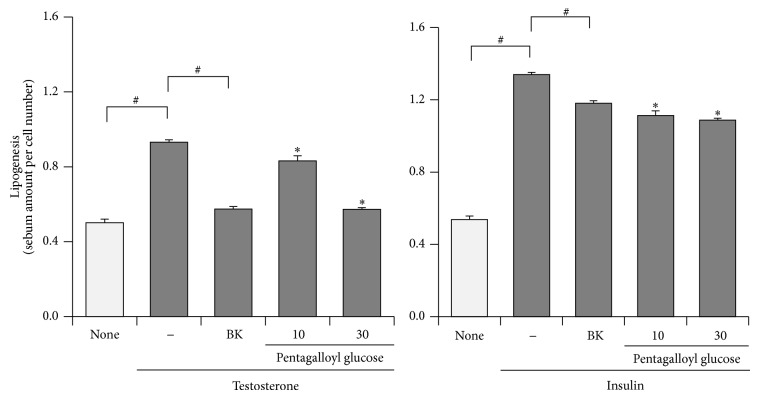
Suppression of testosterone-induced sebum synthesis of hamster sebocytes by pentagalloyl glucose, a main active constituent of BK. Hamster-derived sebaceous gland cells (Ha-SE) were precultured until confluent in the presence of epidermal growth factor and further treated with the indicated concentrations of pentagalloyl glucose or* bokusoku* (BK) (30 *μ*g/mL) and a differentiation factor, 10 *μ*mol/L testosterone or 10 *μ*g/mL insulin. Eight days later, the sebum synthesis was determined using a lipogenesis detecting assay kit. *N* = 3.

Finally, we investigated whether pentagalloyl glucose, the most active and abundant in BK, suppressed testosterone- or insulin-induced lipogenesis in Ha-SE cells. As shown in Figure 5, pentagalloyl glucose significantly downregulated the lipogenesis of both differentiation factors at concentrations of 10 and 30 *μ*mol/L, as well as BK (30 *μ*g/mL) did.

## 4. Discussion

The present study first revealed that BK inhibited 5*α*-reductase activity and testosterone-induced sebum synthesis, a critical factor in the pathogenesis of acne vulgaris. BK contains various types of polyphenols that have been reported to exert multiple biological effects including anti-inflammatory, antiadipotic, antibacterial, and antioxidant activities. Taken together, BK may be useful as an agent to treat acne vulgaris. It should be noted that the gallate polyphenols categorized in the groups of gallotannin and ellagitannin are abundant in BK. In particular, pentagalloyl glucose was identified to be one of the active compounds in BK and strongly reduced lipogenesis.

The compounds marked by an asterisk in Table 1 have been reported to be present in* Quercus acutissima*. However, the pentagalloyl glucoses, eugeniin, 1-desgalloyl eugeniin, and casuarinin, were found in culture fluids of* Quercus acutissima* callus cultures. It is noted that our study is the first to identify these gallate polyphenols in the cortex of* Quercus acutissima,* although they were reported to be present in the cortex of other allied species of* Quercus*. All data reported previously on* Quercus acutissima* indicated qualitative values of constituents. Our study quantified several constituents of BK, which is used as a medical material for treatment of skin diseases.

The main final product of testosterone metabolism in assays using rat liver microsomes was supposed to be 5*α*-androstane-3,17-diol for the following reasons: (1) the mobility of the metabolite was identical to that of its standard, and (2) DHT added to the enzymatic reaction as a substrate was not detected after the enzymatic reaction, and the metabolite appeared at the same relative position as testosterone (Supplementary Figure S2). These results showed that testosterone in this assay was metabolized by 5*α*-reductase to DHT, followed by further conversion to members of 5*α*-androstane-3,17-diol by aldo-keto reductase family 1 (AKR1C1, AKR1C2) [7, 26]. Thus, BK inhibited 5*α*-reductase activity, although DHT itself was not observed due to the lower assay sensitivity. Several studies have indicated testosterone metabolism in sebocytes, showing that the metabolites are DHT, androsterone, androstenedione, 5*α*-androstandione, and 5*α*-androstane-3,17-diol [7, 26]. Therefore, BK may regulate acne pathology depending on testosterone metabolism.

The inhibitory effects of BK and pentagalloyl glucose on sebum synthesis in the intact cells did not depend on their cell cytotoxicity, because cell metabolic activities cultured for 24 h with BK or pentagalloyl glucose in the presence of growth factor EGF were not changed compared with the vehicle control (Supplementary Figure S1), while triton X-100 exerted a significant cytotoxicity. Our study implied that their antilipogenesis depends on suppression of sebaceous differentiation and proliferation in sebocytes rather than their cytotoxicity.

In human SEB-1 sebocytes, insulin activates PI3K/Akt and MAPK/ERK signal transduction pathways and induces the expression of SREBP-1, resulting in increased sebaceous lipogenesis [27]. On the other hand, testosterone and DHT bind to androgen receptors, a type of nuclear receptor, in the cytoplasm, and their complex translocates to the nucleus, leading to up- or downregulation of specific gene transcriptions, including diacylglycerol acyltransferase. It is known that testosterone and DHT augmented the formation of intracellular lipid droplets along with an increase in the accumulation of triglycerides in an assay using hamster sebocytes that was almost the same as that performed in our study [28]. Our study implied BK suppressed testosterone-induced sebum production by blocking the 5*α*-reductase-dependent conversion to the more active androgen DHT. However, further investigation is needed to clarify the action of BK related to 5*α*-reductase.

A handful of* in vitro* and* in vivo* studies have shown that pentagalloyl glucose exhibits several bioactive effects that suggest a great potential for pentagalloyl glucose in the therapy and prevention of several major diseases including benign prostatic hyperplasia, prostate cancer, acne, and androgenic alopecia [29]. In models of cancer and diabetes, pentagalloyl glucose is well studied and indicates promising efficacies. According to a previous report [12], pentagalloyl glucose suppressed rat liver microsomal 5*α*-reductase activity and expression of the androgen receptor in prostate cells, resulting in a significant reduction in androgen-induced prostate cancer cell growth and fatty acid synthase by pentagalloyl glucose.

Our study showed the inhibitory effect of pentagalloyl glucose on the testosterone-dependent cell-based activity, at least partially via regulating 5*α*-reductase activity, because it inhibited testosterone-induced sebum production more effectively than insulin-induced production. However, it is plausible that it also targets other than 5*α*-reductase. For instance, there is a possibility that pentagalloyl glucose directly inhibits enzymatic activity of fatty acid synthase, as reported previously [12, 30]. According to a study of the quantitative structure-activity relationship using gallate catechins, the galloyl moiety is the critical structural feature [31, 32]. Moreover, pentagalloyl glucose is reported to bind to insulin receptors, followed by suppression of insulin functions [33].

It is a wonder if pentagalloyl glucose works on the 5*α*-reductase that is present in the cytoplasm. Pentagalloyl glucose contains five galloyl groups with an ester bond linkage to a core sugar, as shown in Supplementary Figure S3. However, it is expected to pass through the lipid bilayer via a glucose transporter because pentagalloyl glucose crosses a monolayer of human intestinal epithelial Caco-2 cells, and phlorizin, a specific inhibitor of the sodium-dependent glucose transporter SGLT-1, inhibits the transport of pentagalloyl glucose [34, 35].

Other than pentagalloyl glucose, our study found that BK contains various types of gallate polyphenols that may ameliorate skin disorders including acne vulgaris and allergic inflammation. Epigallocatechin gallate is reported to significantly improve acne in humans [15]. Its mechanism was well examined in assays using human sebocytes; that is, epigallocatechin gallate reduces sebum production and inflammation and also induces apoptosis in sebocytes and decreases the viability of* Propionibacterium acnes*. Several papers demonstrated anti-inflammatory and antioxidant effects of epigallocatechin gallate, (+)-taxifolin, gallic acid, and methyl gallate in dermatitis models [29, 36–41]. Neutrophils are recruited to sites of inflammatory rash of acne vulgaris and are activated, leading to production of NADPH oxidase-dependent reactive oxygen species (ROS). The prolonged release of excess ROS in the skin can aggravate inflammatory injury and promote chronic inflammation. It is therefore important to regulate ROS production in acne therapy. Considerable interest has developed in addressing the potential of BK to inhibit ROS production. BK was shown to strongly inhibit superoxygen production by a hypoxanthine-xanthine oxidase system (Supplementary Figure S4).

Current consensus recommends a combination of topical retinoid and antimicrobial therapy as first-line therapy for almost all patients with acne [1, 42]. These medicines were developed to attack directly the skin area affected by acne vulgaris. On the other hand, the pathological changes of acne are significantly affected by diet, psychological stress, and hormonal imbalance, as a systemic disease [43, 44]. Actually, sebocytes express various types of receptors, especially neural and endocrine sensors [4, 5]. That is why acne is recurrent and difficult to cure completely and needs combination therapy with reciprocally different mechanisms and/or features. BK, which inhibits 5*α*-reductase activity and androgen-induced pathogenesis in skin disorders, may be an optional agent in the therapeutic strategy of acne vulgaris.

## Supplementary Material

The Supplementary Material contains 4 figures and 2 tables.
Figure S1: shows that there is no cytotoxic effect of BK and pentagalloyl glucose in hamster primary sebocytes.
Figure S2 presents the bioconversion of exogenous dihydrotestosterone to 5α-androstane-3,17-diol by rat liver microsomal enzymes.
Figure S3 gives the chemical structure of 1,2,3,4,6-penta-O-galloyl glucose (pentagalloyl glucose).
Figure S4 shows the reduction of generation of reactive oxygen species by BK.
Table S1 presents the methods of LC-MS/MS: ion parameters of test compounds.
Table S2 presents the LC-MS/MS Methods: HPLC conditions.

## Figures and Tables

**Table 1:** Quantification of constituents in an extract of BK, from cortex of *Quercus acutissima*.

Compound category	Subcategory	Name	Molecular weight	Amount (mg/g BK)
With gallate group	Gallotannin	Tetragalloyl glucose	788.55	0.767
	Gallotannin	^*^Pentagalloyl glucose	940.68	3.71
	Gallotannin	Hamamelitannin	484.36	0.928
	Ellagitannin	^*^Eugeniin	938.66	4.74
	Ellagitannin	^*^1-Desgalloyl eugeniin	786.54	2.65
	Ellagitannin	^*^Casuarinin	936.64	0.154
	Ellagitannin	^*^Castalagin	934.63	5.91
	Ellagitannin	Stenophyllanin C	1056.14	1.33
	Flavonoid	(−)-Epicatechin gallate	442.37	0.238
	Flavonoid	(−)-Epigallocatechin gallate	458.40	0.0603
		^*^Gallic acid	170.12	2.89
		^*^Methyl gallate	184.15	0.113

Without gallate group	Coumarin	Fraxin	370.31	0.183
	Flavonoid	^*^(+)-Catechin	290.27	10.7
	Flavonoid	^*^(±)-Gallocatechin	306.27	1.69
	Flavonoid	^*^Chrysin	254.24	^†^BQL
	Flavonoid	(+)-Taxifolin	304.25	0.0636
	Flavonoid	Luteolin	286.24	0.00366
	Flavonoid	Quercetin	302.20	0.00856
	Flavonoid	Quercitrin	448.38	0.00142
	Flavonoid	Genistein	270.24	0.000234

Methanol/water extracts of dried BK (Lot No. 2131093010) were analyzed by LC-MS/MS.

^*^Previously reported to exist in the cortex of *Quercus acutissima*.

^†^Amount of chrysin was below the quantification limit (BQL). The limit of quantification of chrysin is 0.000800 mg/g.

**Table 2:** Quantification of inhibitory effect of constituents in extract of BK from cortex of *Quercus acutissima*.

	Inhibitory activity on 5*α*-reductase (% of control)				
BK constituent	Concentration (*μ*mol/L)				IC_50_ (*μ*mol/L)
	0.1	1	10	100	
Tetragalloyl glucose	3.3 ± 0.5	18.7 ± 0.4	63.2 ± 3.0	102.8 ± 1.2	8.1
Pentagalloyl glucose	5.4 ± 1.3	36.1 ± 6.5	83.5 ± 1.3	107.9 ± 2.2	2.5
Eugeniin	4.2 ± 1.0	13.9 ± 0.5	62.5 ± 0.6	114.6 ± 0.6	10.9
1-Desgalloyl eugeniin	4.6 ± 0.2	9.2 ± 1.6	52.5 ± 2.4	107.9 ± 0.9	12.6
Casuarinin	6.4 ± 1.4	10.8 ± 0.7	59.0 ± 2.0	110.1 ± 0.5	10.5
Castalagin	4.9 ± 0.5	6.3 ± 1.2	70.7 ± 3.2	113.3 ± 7.6	8.0
Stenophyllanin C	5.6 ± 0.6	4.6 ± 0.5	63.1 ± 0.4	102.3 ± 2.2	7.2
(−)-Epicatechin gallate	4.6 ± 0.2	4.9 ± 0.2	5.9 ± 0.3	69.4 ± 1.2	10–100
(−)-Epigallocatechin gallate	3.7 ± 0.1	4.4 ± 0.4	5.3 ± 0.3	58.6 ± 2.7	10–100

Testosterone (3.5 *μ*mol/L) was incubated for 30 min in an assay solution containing rat liver microsomes (40 *μ*g/mL), cofactors, and test sample at indicated concentrations. Testosterone remaining in the reaction was extracted with ethyl acetate after incubation and quantified by HPLC. Data are shown as relative amounts of testosterone, which are percentages of the control testosterone alone reaction. *N* = 3. Hamamelitannin, fraxin, gallic acid, methyl gallate, (+)-catechin, (−)-gallocatechin, (+)-gallocatechin, and (+)-taxifolin did not have any effect on 5*α*-reductase activity at a concentration of 100 *μ*mol/L.
