# Supplementary material for: Inhibition of Rat 5α-Reductase Activity and Testosterone-Induced Sebum Synthesis in Hamster Sebocytes by an Extract of Quercus acutissima Cortex
Source: Evid Based Complement Alternat Med. 2015 Jan 29;2015:853846. doi: 10.1155/2015/853846 (PMC4325551; doi:10.1155/2015/853846)
Supplement: Supplementary file 1 — The Supplementary Material contains 4 figures and 2 tables. Figure S1: shows that there is no cytotoxic effect of BK and pentagalloyl glucose in hamster primary sebocytes. Figure S2 presents the bioconversion of exogenous dihydrotestosterone to 5α-androstane-3,17-diol by rat liver microsomal enzymes. Figure S3 gives the chemical structure of 1,2,3,4,6-penta-O-galloyl glucose (pentagalloyl glucose). Figure S4 shows the reduction of generation of reactive oxygen species by BK. Table S1 presents the methods of LC-MS/MS: ion parameters of test compounds. Table S2 presents the LC-MS/MS Methods: HPLC conditions. [file 853846.f1.pdf]

Supplementary data

Four Figures and Two Tables

Fig. S1. No cytotoxic effect of BK and pentagalloyl glucose in hamster primary sebocytes

Hamster-derived sebaceous gland cells (Ha-SE) were precultured until confluent in the presence of 10 ng/mL epidermal growth factor (EGF), and further cultured with 10, 30 μg/mL *bokusoku* (BK), 10, 30 μmol/L pentagalloyl glucose, 0.2% triton X-100, or vehicle in the presence of EGF. Twenty four hours later, cell cytotoxicity was measured using a XTT reagent. N=3

Fig. S2. Bioconversion of exogenous dihydrotestosterone to 5α-androstane-3,17-diol by rat liver microsomal enzymes

Dihydrotestosterone (DHT) was incubated at a final concentration of 3.5 μmol/L for 30 min in the presence or absence of rat liver microsomes (40 μg/mL) and the co-factors. The metabolites were extracted with ethyl acetate after incubation, and analyzed by TLC. All androgen standards were applied at 0.1 μg/μL/spot. T.: testosterone, DHT: dihydrotestosterone, A.diol: 5α-androstane-3α,17β-diol

Fig. S3. Chemical structure of 1,2,3,4,6-penta-O-galloyl glucose (pentagalloyl glucose)

Fig. S4. Reduction of generation of reactive oxygen species by BK

Chemiluminescent assay for determining xanthine oxidase activity was performed using a superoxide probe of 2-methyl-6-p-methoxyphenylethynylimidazopyrazinone (MPEC; ATTO Co., Tokyo, Japan). In brief, *bokusoku* (BK) or ascorbic acid was added at the indicated concentrations to the reaction containing hypoxanthine, xanthinoxidase from buttermilk (Oriental Yeast Co., Tokyo, Japan), MPEC. Superoxide generated in the reaction for 1 min was evaluated by measuring chemiluminescent intensity. N=3,

Supplementary Table S1. Methods of LC-MS/MS: Ion parameters of test compounds

Compound name	Q1Mass (*m/z*)	Q3Mass (*m/z*)	DP (volts)	CE (volts)	CXP (volts)	#
tetragalloyl glucose	787.213	168.9	-155	-70	-13	1-1
pentagalloyl glucose	939.262	769.1	-165	-44	-21	1-1
hamamelitannin	483.18	168.8	-105	-42	-11	1-1
eugeniin	937.241	301	-175	-64	-17	1-1
1-desgalloyl eugeniin	785.268	300.7	-175	-56	-19	1-1
(-)-epicatechin gallate	441.043	168.9	-80	-28	-13	1-2
(-)-epigallocatechin gallate	457.151	124.8	-95	-54	-19	1-2
(+)-catechin	288.917	109.1	-90	-32	-17	1-2
(±)-gallocatechin	305.057	125.1	-95	-30	-19	1-2
chrysin	252.93	142.8	-80	-38	-9	1-2
luteolin	284.85	132.3	-90	-68	-23	1-2
quercetin	300.98	150.7	-105	-30	-9	1-2
quercitrin	447.103	300.5	-110	-36	-17	1-2
genistein	268.919	132.7	-105	-42	-21	1-2
gallic acid	168.79	125	-65	-22	-19	1-3
methyl gallate	182.851	123.9	-65	-30	-19	1-3
casuarinin	937.096	345.1	181	39	24	2-4
castalagin	935.025	468.9	181	39	22	2-4
stenophyllanin C	1057.252	585.2	216	43	10	2-4
fraxin	371.07	209.1	31	19	12	2-4
(+)-taxifolin	305.071	149.1	71	31	8	2-4

Limits of quantification were 40 ng/mL for tetragalloyl glucose and 1-desgalloyl eugeniin; 3200 ng/mL for pentagalloyl glucose; 4 ng/mL for hamamelitannin and methyl gallate; 1600 ng/mL for eugeniin; 10 ng/mL for (-)-epicatechin gallate, chrysin, luteolin, quercetin, quercitrin, fraxin, and (+)-taxifolin; 100 ng/mL for (-)-epigallocatechin gallate, (+)-catechin, (±)-gallocatechin, casuarinin, and castalagin; 2 ng/mL for genistein; 200 ng/mL for gallic acid and stenophyllanin C.

#: LC-MS/MS system and HPLC method ID described in supplementary Table S2.

DP: declustering potential

CE: collision energy

CXP: collision cell exit potential

Supplementary Table S2. LC-MS/MS Methods:HPLC Conditions

LC-MS/MS System	HPLC method	HPLC conditions
1	1	**Column:** Ascentis Express RP-amide column (100 × 2.1 mm ID, 2.7-μm particle size; Supelco Analytical, Inc., Tokyo, Japan)<br>**Mobile phase:** (A) 0.2 vol % acetic acid, (B) acetonitrile containing 0.2 vol % acetic acid<br>**Gradient elution program (% B in A):** 0-8 min, 22%; 8-8.01 min, 22-90%; 8.01-13 min, 90%; 13-13.01 min, 90-22%; 13.01-18 min, 22%<br>Other conditions: flow rate, 0.2 mL/min; column temperature, 40°C; injection volume, 10 μL
	2	**Column:** Ascentis Express RP-amide column<br>**Mobile phase:** (A) 0.2 vol % acetic acid, (B) acetonitrile containing 0.2 vol % acetic acid<br>**Gradient elution program (% B in A):** 0-5 min, 22%; 5-10 min, 22-90%; 10-15 min, 90%; 15-15.1 min, 90-22%; 15.1-25 min; 22%<br>Other conditions: flow rate, 0.2 mL/min; column temperature, 40°C; injection volume, 10 μL
	3	**Column:** Ascentis Express RP-amide column<br>**Mobile phase:** (A) 0.2 vol % acetic acid, (B) acetonitrile containing 0.2 vol % acetic acid<br>**Gradient elution program (% B in A):** 0-2 min, 22%; 2-10 min, 22-80%; 10-15 min, 80%; 15-15.1 min, 80-22%; 15.1-20 min, 22%<br>Other conditions: flow rate, 0.2 mL/min; column temperature, 40°C; injection volume, 10 μL
2	4	**Column:** Ascentis Express RP-amide column<br>**Mobile phase:** (A) 0.2 vol % formic acid, (B) acetonitrile<br>**Gradient elution program (% B in A):** 0-1 min, 10%; 1-8 min, 10-30%; 8-10 min, 30-90%, 10-12 min, 90%; 12-12.01 min, 90-10 %;12.01-17 min, 10%<br>Other conditions: flow rate, 0.3 mL/min; column temperature, 40°C; injection volume, 10 μL

LC-MS/MS system: system 1, an API4000 triple quadrupole mass spectrometer (AB SCIEX, Tokyo, Japan) equipped with an Agilent 1100 system (Agilent Technologies, Inc., Tokyo, Japan); system 2, a TripleQuad6500® (AB SCIEX) equipped with an Agilent 1290 system (Agilent Technologies, Inc.)

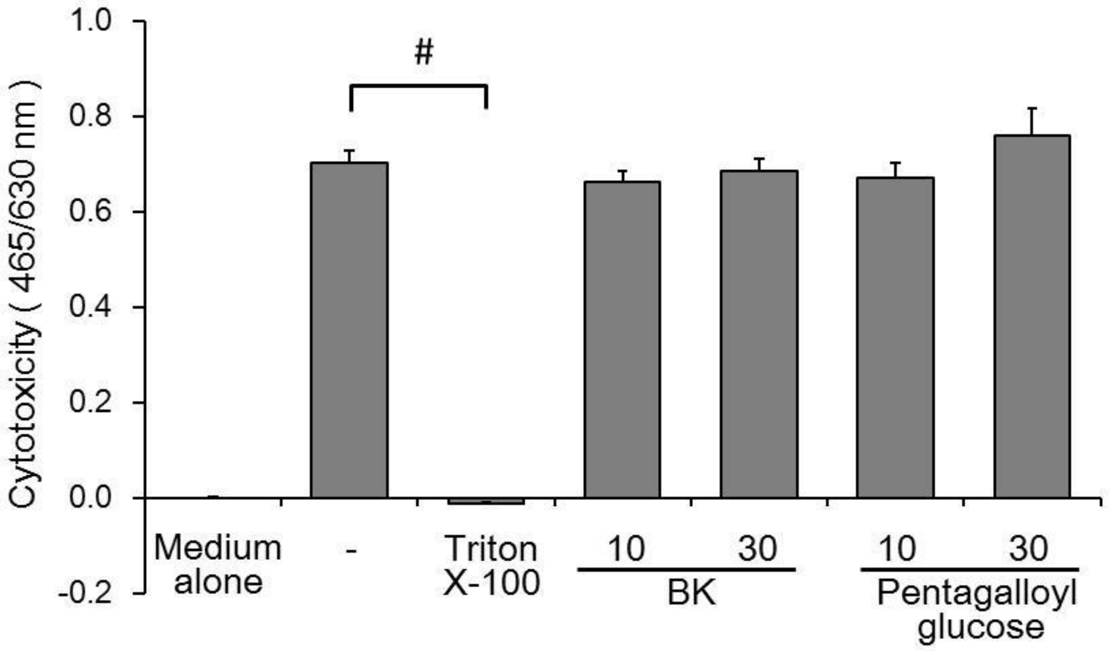
1.0
0.8
0.6
0.4
0.2
0.0
-0.2
Cytotoxicity (465/630 nm)
#
Medium alone
-
Triton X-100
10
30
BK
10
30
Pentagalloyl glucose

Fig. S1

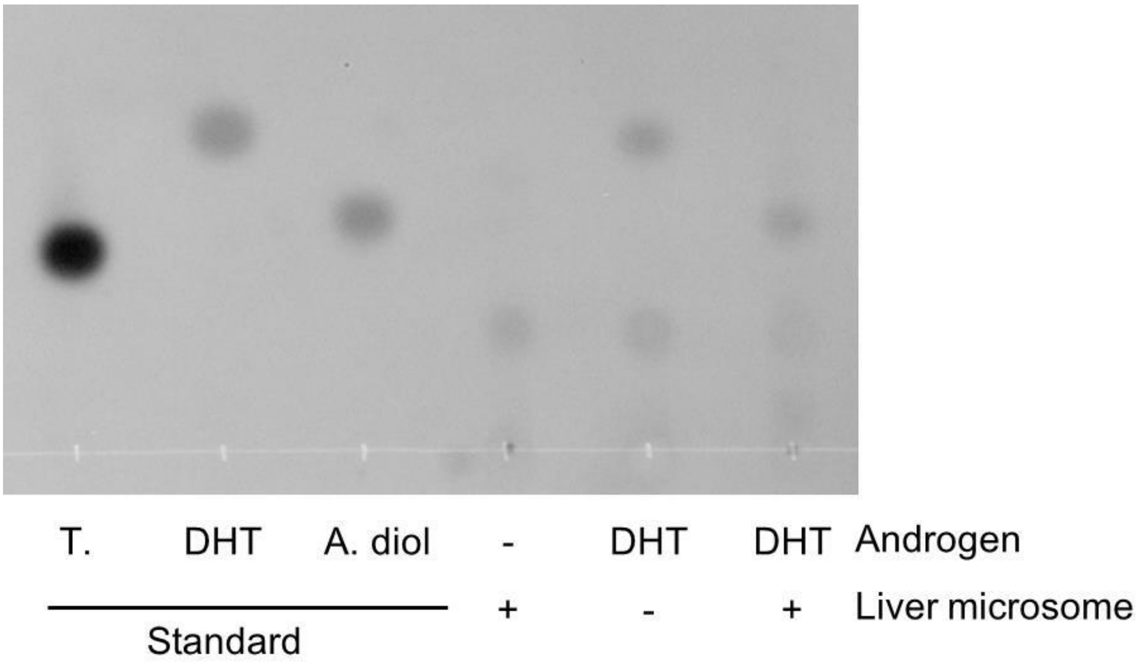

Fig. S2

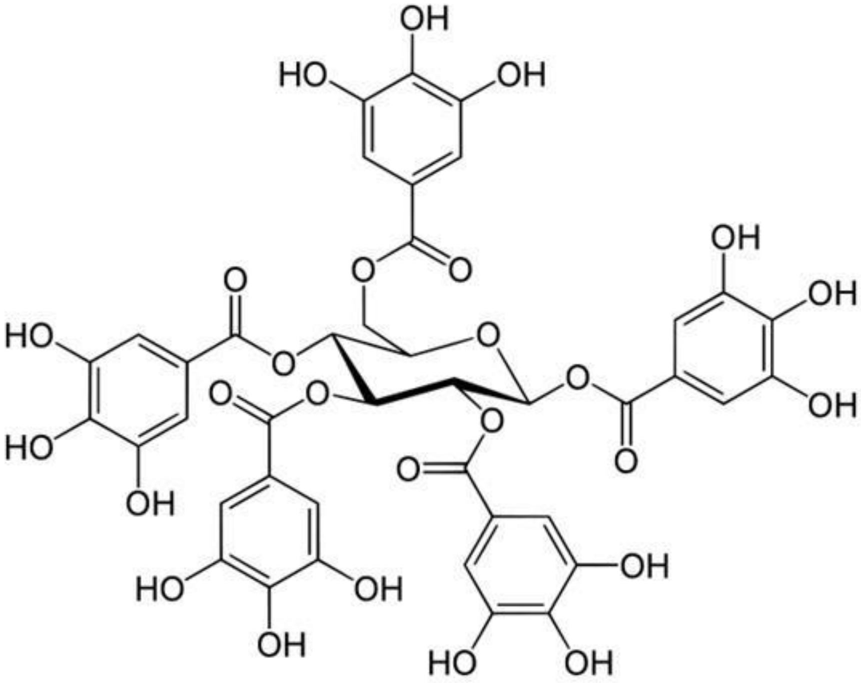

Fig. S3

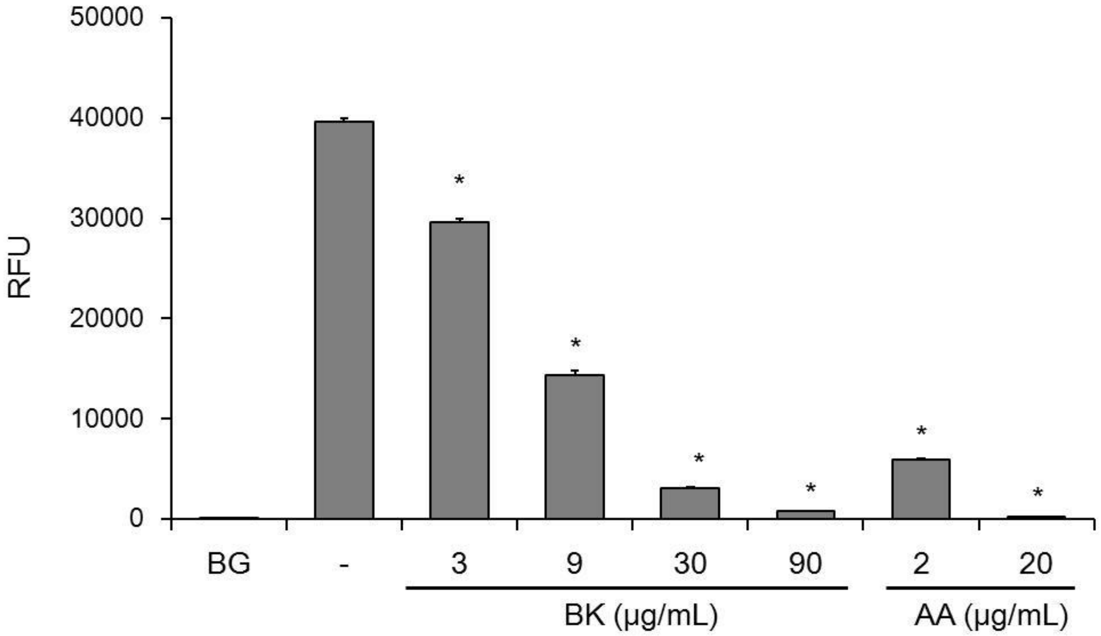
50000
40000
30000
20000
10000
0
RFU
*
*
*
*
*
*
BG
-
3
9
30
90
2
20
BK (μg/mL)
AA (μg/mL)

Fig. S4
